# Supplementary material for: More than Just a Shell: Indehiscent Fruits Drive Drought-Tolerant Germination in Invasive Lepidium Species
Source: Plants (Basel). 2025 May 19;14(10):1517. doi: 10.3390/plants14101517 (PMC12115056; doi:10.3390/plants14101517)
Supplement: Supplementary file 1 [file plants-14-01517-s001.zip › plants-3620574-supplementary.pdf]

Supplemental Material

Supplemental Table S1. Mean germination (%) and ANOVA tests for various drought stress treatments: (A) *Lepidium appelianum* fresh fruits and manually isolated seeds , (B) *Lepidium draba* fresh and after-ripened fruits, fresh seeds (manually isolated), (C) *Lepidium campestre* fresh and after-ripened seeds. Data underwent one-way ANOVA, followed by post hoc comparisons using Tukey's honest significant difference test. The significance threshold for all analyses was set at P < 0.05.

A. *Lepidium appelianum*

Diaspore	MPa	N	Germination (%) Mean ± SE
Fresh fruits	-0.8	3	38±1.7
	-0.6	3	52±1.6
	-0.4	3	80±1.15
	-.2	3	83.3±0.8
	0.0	3	88.3±1.6
Fresh seeds	-0.8	3	36±1.2
	-0.6	3	52.67±1.8
	-0.4	3	78.3±1.6
	-.2	3	82.3±1.4
	0.0	3	86.67±1.6

ANOVA

		Sum of Squares	Df	Mean Square	F	Sig.
Fresh fruits	Between Groups	5844.000	4	1461.000	322.279	<.001
	Within Groups	45.333	10	4.533		
	Total	5889.333	14			
Fresh seeds	Between Groups	5749.733	4	1437.433	197.812	<.001
	Within Groups	72.667	10	7.267		
	Total	5822.400	14			

B. *Lepidium draba*

Diaspore	MPa	N	Germination (%) Mean ± SE
Fresh fruits	-0.8	3	0.0±0
	-0.6	3	14±1.1
	-0.4	3	17.3±1.3
	-.2	3	42.3±1.4

	**0.0**	**3**	56±2.3
**After-ripened fruits**	**-0.8**	**3**	0.0±0
	**-0.6**	**3**	20±1.5
	**-0.4**	**3**	47.3±4.3
	**-.2**	**3**	53.3±3.7
	**0.0**	**3**	86.67±1.6
**Fresh seeds**	**-0.8**	**3**	0.0±0
	**-0.6**	**3**	8.67±1.7
	**-0.4**	**3**	45.3±0.6
	**-.2**	**3**	53.67±1.2
	**0.0**	**3**	86.67±1.6

ANOVA

		Sum of Squares	Df	Mean Square	F	Sig.
Fresh fruits	Between Groups	6185.600	4	1546.400	244.168	<.001
	Within Groups	63.333	10	6.333		
	Total	6248.933	14			
After-ripened fruits	Between Groups	13195.733	4	3298.933	148.601	<.001
	Within Groups	222.000	10	22.200		
	Total	13417.733	14			
Fresh seeds	Between Groups	14905.067	4	3726.267	798.486	<.001
	Within Groups	46.667	10	4.667		
	Total	14951.733	14			

C. *Lepidium campestre*

Diaspore	MPa	N	Germination (%) Mean ± SE
**Fresh seeds**	**-0.8**	**3**	0.0±0
	**-0.6**	**3**	0.0±0
	**-0.4**	**3**	6±1.2
	**-.2**	**3**	18±1.1
	**0.0**	**3**	34.3±1.4

**After-ripened seeds**	**-0.8**	**3**	0.0±0
	**-0.6**	**3**	9.3±3.5
	**-0.4**	**3**	34.6±1.7
	**-.2**	**3**	46±1.4
	**0.0**	**3**	86.6±1.6

ANOVA

		Sum of Squares	Df	Mean Square	F	Sig.
Fresh seeds	Between Groups	2574.667	4	643.667	224.535	<.001
	Within Groups	28.667	10	2.867		
	Total	2603.333	14			
After-ripened seeds	Between Groups	14021.333	4	3505.333	297.062	<.001
	Within Groups	118.000	10	11.800		
	Total	14139.333	14			
